# Retraction Note: Comparing ANI-2x, ANI-1ccx neural networks, force field, and DFT methods for predicting conformational potential energy of organic molecules

**DOI:** 10.1038/s41598-026-42379-1

**Published:** 2026-03-02

**Authors:** Mozafar Rezaee, Saeid Ekrami, Seyed Majid Hashemianzadeh

**Affiliations:** 1 Molecular Simulation Research Laboratory, Department of Chemistry, Iran University of Science and Technology, Tehran, Iran; 2 CNRS, LCPME, Université de Lorraine, 54000 Nancy, France

Retraction of: *Scientific Reports* 10.1038/s41598-024-62242-5, published online 23 May 2024

The Editors have retracted this Article.

After publication, concerns about the validity of the results reported in the Article were brought to the attention of the Editors, who requested the raw data underlying the study, but the Authors were unable to provide those. The Editors no longer have confidence in the reliability of the Article.

All authors disagree with this retraction.

